# AI-driven digital twins for bone tumors: personalizing immunoradiotherapy and drug-based radiosensitization

**DOI:** 10.3389/fphar.2026.1789971

**Published:** 2026-03-26

**Authors:** Jian Tong, Daoyu Chen, Haobo Chen, Tao Yu

**Affiliations:** Department of Spinal Surgery, No. 1 Orthopedics Hospital of Chengdu, Chengdu, China

**Keywords:** artificial intelligence, bone tumors, digital twin(s), immunoradiotherapy, radiosensitization, radiotherapy planning

## Introduction

Primary malignant bone tumors—such as osteosarcoma, Ewing sarcoma, and chondrosarcoma—are clinically challenging because they are biologically heterogeneous, relatively rare, and often arise in sites where local control must be balanced against long-term toxicity and function. Treatment is multimodal, and many radiotherapy and systemic therapy decisions are therefore trade-off driven. Meanwhile, immunotherapy for bone tumors remains difficult to generalize across subtypes and patients, motivating continued method development and better patient selection (Yu and Yao, 2024; Wang et al., 2025).

A digital twin has been proposed as a patient-specific computational representation that can be updated with longitudinal data to forecast trajectories under competing interventions. In oncology, the digital twin vision is explicitly tied to counterfactual experimentation: using a model to ask “what if we change schedule, dose, or combination?” rather than merely predicting outcomes under usual care (Hernandez-Boussard et al., 2021; Katsoulakis et al., 2024). Health digital twin reviews also emphasize the importance of clearly defining intended use, data flows, and validation plans so that digital twins become decision-support systems rather than abstract metaphors (Katsoulakis et al., 2024; Tudor et al., 2025). From a complex-systems perspective, digital twins in precision medicine face challenges in scalability, interpretability, and reliable personalization across heterogeneous settings (De Domenico et al., 2025).

This Opinion advances the viewpoint that bone tumors are a suitable proving ground for oncology digital twins, provided the twins are narrowly scoped to actionable decisions and developed with explicit verification, validation, and uncertainty quantification. We focus on two use cases where current practice faces persistent uncertainty: (i) personalizing immunoradiotherapy (immunotherapy combined with radiotherapy) and (ii) rationally selecting drug-based radiosensitizers.

### What should count as a bone-tumor digital twin?

Health-oriented definitions converge on several core properties: a digital twin should be individualized, dynamically updated with new observations, and capable of evaluating counterfactual interventions rather than only forecasting an observed trajectory (Katsoulakis et al., 2024; Tudor et al., 2025). In cancer, mechanistic plausibility is important because treatment choices manipulate biology. Accordingly, digital twin proposals in immuno-oncology frequently highlight mechanistic quantitative systems pharmacology (QSP) as a foundation for simulating therapy perturbations (Wang et al., 2024).

For bone tumors, a pragmatic definition is a hybrid model that (i) ingests routine clinical data (imaging, radiotherapy plans and delivery, pathology and staging, and standard laboratory values), (ii) contains interpretable modules for radiobiology and immune–tumor interaction, (iii) is recalibrated during treatment at major clinical milestones (e.g., mid-course MRI or scheduled blood draws) as new observations arrive, and (iv) outputs decision-relevant probability distributions rather than point predictions. Machine learning (ML) is used to personalize parameters and capture residual patterns, while mechanistic modules constrain extrapolation to clinically meaningful counterfactuals (Wang et al., 2024; Katsoulakis et al., 2024).

### Why immunoradiotherapy in bone tumors is an appropriate first target

Radiotherapy can reshape the tumor immune microenvironment through immunogenic cell death, antigen release, changes in antigen presentation, and altered immune trafficking, creating a rationale for synergy with immunotherapy (Zhang et al., 2022; Guo et al., 2023). However, sarcoma-focused reviews stress that radiotherapy–immunotherapy combinations remain difficult to optimize because key design variables—dose, fractionation, target volume, and sequencing—are likely context dependent and hard to test exhaustively in bone tumors (Zhang et al., 2024).

These characteristics match the strengths of a digital twin. Many immunoradiotherapy decisions are inherently counterfactual (e.g., concurrent versus sequential immune checkpoint inhibition relative to radiotherapy, or whether to intensify local dose to favor systemic immune priming), yet practical constraints limit the number of regimens that can be evaluated prospectively in bone tumors. Moreover, immunoradiotherapy efficacy likely depends on patient-specific immune activation and suppression dynamics, including myeloid-driven immune suppression and T cell exclusion (Zhang et al., 2024).

### A minimum viable immunoradiotherapy twin for bone tumors

A clinically tractable twin should begin from data streams already embedded in care. A medical-imaging review describes how imaging can anchor digital twin construction through segmentation, longitudinal tracking, and response modeling across computed tomography (CT), magnetic resonance imaging (MRI), and positron emission tomography (PET) when available (Zhao et al., 2025). These data naturally couple to radiotherapy planning, which provides three-dimensional dose distributions and treatment timing.

We propose four modules as a minimum viable bone-tumor immunoradiotherapy twin: (1) Tumor–dose geometry: mapping tumor burden and spatial extent to delivered dose, with uncertainty reflecting contouring and delivery variability (Zhao et al., 2025). (2) Radiobiology: individualized parameters for cell kill and regrowth, updated using early imaging response rather than fixed population values, aligning with the “predictive oncology” digital twin vision (Hernandez-Boussard et al., 2021; Zhao et al., 2025). (3) Immune dynamics: a simplified representation of antigen release, priming, effector trafficking, and suppressive myeloid programs, calibrated to peripheral blood markers and, where feasible, tumor immune profiling (Guo et al., 2023; Wang et al., 2024). (4) Decision and uncertainty layer: simulation of alternative schedules and reporting of outcome distributions consistent with verification, validation, and uncertainty quantification principles (Sel et al., 2025).

Crucially, implementing this in rare, heterogeneous bone tumors inevitably encounters a ‘cold start’ problem at diagnosis. Before longitudinal data is available, relying solely on population-based priors risks early inaccuracies. To mitigate this, the twin must utilize stratified baseline priors (e.g., multi-omics and radiomics) and explicitly communicate this initial state through wide uncertainty distributions. Once early longitudinal observations arrive, the model rapidly updates these priors into individualized posteriors.

The output should be concrete. For example, the twin could compare concurrent versus sequential immune checkpoint inhibition relative to radiotherapy, estimate a probability distribution of local control and immune-mediated systemic response under each option, and recommend which additional biomarker (e.g., a serial immune subset or inflammatory marker) would most reduce uncertainty before committing to a schedule. This “value of information” framing aligns with the argument that digital twins should guide not only decisions, but also data collection that makes decisions safer (Katsoulakis et al., 2024; Sel et al., 2025).

### Digital twins to rationalize drug-based radiosensitization

Drug-based radiosensitization aims to increase tumor response to radiotherapy by targeting biological processes that confer radioresistance, but translation has been constrained by toxicity and uncertain patient selection. Poly (ADP-ribose) polymerase (PARP) inhibitors have been highlighted as radiosensitizers that can amplify radiation-induced DNA damage, motivating careful exploration of combined-modality schedules and safety (Sun et al., 2023). Preclinical work in osteosarcoma has also explored olaparib as a radiosensitizer with photons and carbon ions (Dong et al., 2024). Sarcoma-specific clinical exploration is emerging; for example, a phase Ib study evaluated olaparib with concomitant radiotherapy in soft-tissue sarcoma (Sargos et al., 2025).

A digital twin can make radiosensitization more “mechanism matched” by linking drug exposure (pharmacokinetics/pharmacodynamics), DNA repair capacity, and microenvironmental state to radiotherapy response. Mechanistic modules can encode DNA damage response and repair kinetics, while ML layers use imaging and molecular correlates to personalize these parameters and quantify uncertainty (Wang et al., 2024; Zhao et al., 2025). The twin’s output should be risk informed: rather than recommending a radiosensitizer broadly, it should estimate the probability of benefit and toxicity under candidate schedules and identify the patient features that drive uncertainty, thereby prioritizing who should enter combination trials and under what monitoring plans (Sel et al., 2025).

### Incorporating emerging radiosensitization mechanisms: ferroptosis as an example

Ferroptosis has been reviewed as a regulated cell-death modality relevant to radiotherapy and combination strategies, suggesting opportunities to exploit redox vulnerabilities alongside radiation (Lei et al., 2021). In osteosarcoma, synergistic pathway inhibition has been reported to sensitize tumor cells to ionizing radiation by inducing ferroptosis, providing an example of a mechanistically distinct radiosensitization route (Xiao et al., 2024). Such findings do not immediately justify clinical deployment, but they provide testable hypotheses for patient selection and combination design.

From a digital twin perspective, the key is evidence gating and modularity. Ferroptosis can be introduced as a latent susceptibility parameter that modulates radiation response in a subset of patients, with priors informed by emerging biomarkers and updated as data become available (Lei et al., 2021; De Domenico et al., 2025). The twin can then prioritize which mechanistic hypotheses merit prospective collection of specific redox-related markers or window-of-opportunity studies, reducing the risk that promising mechanisms remain stranded in preclinical space.

### Trustworthiness and governance: verification, validation, and uncertainty quantification

Clinical adoption will be bottlenecked by trust rather than model sophistication. A 2025 perspective argues that verification (correct implementation), validation (adequacy for intended use), and uncertainty quantification must be explicit and aligned with the twin’s intended use, because the same model may be adequate for one decision but unsafe for another (Sel et al., 2025). Health digital twin reviews similarly emphasize that inconsistent definitions and weak validation practices have limited real-world impact (Katsoulakis et al., 2024; Tudor et al., 2025).

We therefore advocate staged evaluation for bone-tumor digital twins: (i) technical verification and reproducibility testing, (ii) retrospective validation across multiple institutions with sensitivity analyses for data shift, (iii) prospective “shadow mode” operation to measure calibration drift and data requirements, and (iv) prospective interventional evaluation focused on decision quality and patient outcomes, not only predictive accuracy (Sel et al., 2025). Governance should include transparent reporting of failure modes, explicit uncertainty communication, and monitoring for subgroup performance disparities, because rare cancers are particularly vulnerable to hidden biases from small, non-representative datasets (Tudor et al., 2025).

### Making digital twins testable: trial designs for bone tumors

Sarcoma immunoradiotherapy reviews identify dose, fractionation, sequencing, and patient selection as unresolved variables that are naturally testable and potentially optimizable (Zhang et al., 2024). This enables evaluation designs where the twin itself is the intervention. One practical pathway is a two-stage approach: develop a twin that outputs a small menu of protocolized regimens with uncertainty estimates, then evaluate twin-informed decision support against standard protocols using pragmatic or adaptive trial designs that emphasize feasibility and safety (Sel et al., 2025).

Bone tumor care already relies on multidisciplinary tumor boards. A twin can therefore be framed as a structured way to quantify counterfactual reasoning rather than an automated “chooser,” which aligns with adoption guidance in health digital twin reviews (Katsoulakis et al., 2024; Tudor et al., 2025). Even modest success—reliably identifying when schedule A is preferable to schedule B for a defined subgroup—would represent meaningful clinical progress and could accelerate evidence generation in a field constrained by low incidence.

## Discussion

Bone tumors offer a high-value domain to demonstrate what oncology digital twins can and cannot do. The field already produces rich longitudinal imaging and radiotherapy data, and current evidence supports plausible radiotherapy–immunotherapy synergy while also highlighting regimen heterogeneity and patient selection challenges (Zhang et al., 2022; Guo et al., 2023; Zhang et al., 2024). Meanwhile, recent syntheses of healthcare digital twins stress that clinical impact requires purpose-built models with clear intended use, transparent evaluation, and uncertainty-aware outputs (Katsoulakis et al., 2024; Tudor et al., 2025).

Our viewpoint is deliberately incremental. The first-generation bone-tumor digital twin should not attempt to simulate the entire disease course. Instead, it should target a narrow, high-frequency decision (e.g., immunoradiotherapy sequencing, or whether to add a radiosensitizer such as a PARP inhibitor), and it should be evaluated for that intended use with staged verification, validation, and uncertainty quantification (Sel et al., 2025). If developed and tested in this disciplined manner, artificial intelligence–enabled digital twins could become a practical framework to accelerate learning and personalize therapy in bone tumors—particularly for immunoradiotherapy optimization and drug-based radiosensitization—while making uncertainty visible and decision-making more transparent.

## References

[B1] De Domenico M. Allegri L. Caldarelli G. d'Andrea V. Di Camillo B. Rocha L. M. (2025). Challenges and opportunities for digital twins in precision medicine from a complex systems perspective. NPJ Digit. Med. 8 (1), 37. 10.1038/s41746-024-01402-3 39825012 PMC11742446

[B2] Dong M. Luo H. Liu R. Zhang J. Yang Z. Wang D. (2024). Radiosensitization of osteosarcoma cells using the PARP inhibitor olaparib combined with X-rays or carbon ions. J. Cancer 15 (3), 699–713. 10.7150/jca.90371 38213724 PMC10777037

[B3] Guo S. Yao Y. Tang Y. Xin Z. Wu D. Ni C. (2023). Radiation-induced tumor immune microenvironments and potential targets for combination therapy. SIGNAL Transduct. TAR 8 (1), 205. 10.1038/s41392-023-01462-z 37208386 PMC10199044

[B4] Hernandez-Boussard T. Macklin P. Greenspan E. J. Gryshuk A. L. Stahlberg E. Syeda-Mahmood T. (2021). Digital twins for predictive oncology will be a paradigm shift for precision cancer care. Nat. Med. 27 (12), 2065–2066. 10.1038/s41591-021-01558-5 34824458 PMC9097784

[B5] Katsoulakis E. Wang Q. Wu H. Shahriyari L. Fletcher R. Liu J. (2024). Digital twins for health: a scoping review. NPJ Digit. Med. 7 (1), 77. 10.1038/s41746-024-01073-0 38519626 PMC10960047

[B6] Lei G. Mao C. Yan Y. Zhuang L. Gan B. (2021). Ferroptosis, radiotherapy, and combination therapeutic strategies. PROTEIN CELL 12 (11), 836–857. 10.1007/s13238-021-00841-y 33891303 PMC8563889

[B7] Sargos P. Sunyach M. P. Ducassou A. Llacer C. Dinart D. Michot A. (2025). Results of a phase Ib study of olaparib with concomitant radiotherapy in soft-tissue sarcoma: a French sarcoma group study. Ann. Oncol. 36 (5), 592–600. 10.1016/j.annonc.2025.01.016 39894354

[B8] Sel K. Hawkins-Daarud A. Chaudhuri A. Osman D. Bahai A. Paydarfar D. (2025). Survey and perspective on verification, validation, and uncertainty quantification of digital twins for precision medicine. NPJ Digit. Med. 8 (1), 40. 10.1038/s41746-025-01447-y 39825103 PMC11742391

[B9] Sun C. Chu A. Song R. Liu S. Chai T. Wang X. (2023). PARP inhibitors combined with radiotherapy: are we ready? Front. Pharmacol. 14, 1234973. 10.3389/fphar.2023.1234973 37954854 PMC10637512

[B10] Tudor B. H. Shargo R. Gray G. M. Fierstein J. L. Kuo F. H. Burton R. (2025). A scoping review of human digital twins in healthcare applications and usage patterns. NPJ Digit. Med. 8 (1), 587. 10.1038/s41746-025-01910-w 41028914 PMC12484800

[B11] Wang H. Arulraj T. Ippolito A. Popel A. S. (2024). From virtual patients to digital twins in immuno-oncology: lessons learned from mechanistic quantitative systems pharmacology modeling. NPJ Digit. Med. 7 (1), 189. 10.1038/s41746-024-01188-4 39014005 PMC11252162

[B12] Wang C. Chen Z. Mo H. Wang J. Zhou W. (2025). A review of immunotherapy for bone tumors. Front. Immunol. 16, 16. 10.3389/fimmu.2025.1580598 41142821 PMC12545153

[B13] Xiao H. Jiang N. Zhang H. Wang S. Pi Q. Chen H. (2024). Inhibitors of APE1 redox and ATM synergistically sensitize osteosarcoma cells to ionizing radiation by inducing ferroptosis. Int. Immunopharmacol. 139, 112672. 10.1016/j.intimp.2024.112672 39032469

[B14] Yu S. Yao X. (2024). Advances on immunotherapy for osteosarcoma. Mol. Cancer 23 (1), 192. 10.1186/s12943-024-02105-9 39245737 PMC11382402

[B15] Zhang Z. Liu X. Chen D. Yu J. (2022). Radiotherapy combined with immunotherapy: the dawn of cancer treatment. SIGNAL Transduct. TAR 7 (1), 258. 10.1038/s41392-022-01102-y 35906199 PMC9338328

[B16] Zhang Q. S. Hayes J. P. Gondi V. Pollack S. M. (2024). Immunotherapy and radiotherapy combinations for sarcoma. Semin. Radiat. Oncol. 34 (2), 229–242. 10.1016/j.semradonc.2023.12.005 38508787

[B17] Zhao F. Wu Y. Hu M. Chang C. W. Liu R. Qiu R. (2025). Current progress of digital twin construction using medical imaging. J. Appl. Clin. Med. Phys. 26 (9), e70226. 10.1002/acm2.70226 40841176 PMC12370374

